# Genes associated with epithelial mesenchymal transition (EMT) in cervical cancer progression

**DOI:** 10.1007/s12672-025-04011-y

**Published:** 2025-11-20

**Authors:** Kiran Kumari, Raviranjan Kumar Gupta, Saket Kumar, Shyam Babu Prasad

**Affiliations:** 1 Cancer Genomics and Therapeutics Laboratory, Department of Zoology, School of Life Sciences, Mahatma Gandhi Central University Bihar (MGCUB), Motihari, 845401 India; 2 Department of Surgical Gastroenterology, Indira Gandhi Institute of Medical Sciences (IGIMS), Sheikhpura, Patna India

**Keywords:** Cervical cancer (CC), Epithelial-Mesenchymal transition (EMT), Signaling, Gene ontology, Protein–Protein interaction (PPI), Hub genes, Survival analysis.

## Abstract

Cervical Cancer is the second most common gynecological malignancy affecting a large group of women worldwide. The molecular mechanism of cervical cancer progression is still not very clear. As a result, diagnosis of cervical cancer occurs at a very advanced stage when the disease has spread to its malignant stage, causing death in the majority of women. EMT is a major culprit associated with the malignant transformation of tumor cells during cancer progression and metastasis. Hence, identification of new biomarkers to detect cervical cancer at an early stage is essential to minimize incidence and mortality. The present study aims to identify Common Differentially Expressed Genes (DEGs) and early biomarkers associated with EMT in cervical cancer. The Datasets were downloaded from the Gene Expression Omnibus (GEO) database, with Accession numbers GSE26511, GSE67522, and GSE9750. Then, the Gene Ontology (GO), KEGG pathway enrichment analysis, and protein–protein interactions (PPI) were done. Further hub genes were identified by molecular interaction networks using Cytoscape from the constructed network of DEGs. Afterwards, survival analysis was performed to assess the prognostic significance of eight hub genes associated with EMT in cervical cancer. A total of 11,339 overlapping DEGs were identified from all three datasets, among all the total 61 DEGS, and 8 hub genes were linked to the EMT pathway. Our study suggests that these eight hub genes, CDH1, CDH2, MMP2, CD44, FN1, FGF2, SNAI1, and SNAI2, may be critically associated with EMT progression. Among the eight identified EMT hub genes, CDH2 (N-cadherin) demonstrated a significant association with overall survival, while FN1 (fibronectin) was notably linked to disease-free survival, underscoring their prognostic value in cervical cancer. Based on these findings, our study suggests that CDH1, CDH2, MMP2, CD44, FN1, FGF2, SNAI1, and SNAI2 hold potential diagnostic and prognostic significance in the progression of cervical cancer.

**Supplementary Information:**

The online version contains supplementary material available at 10.1007/s12672-025-04011-y.

## Introduction

 Cancer of the Uterine Cervix, called Cervical Cancer (CC), is the second most common cancer in women worldwide, with 604,000 new cases in 2022 (Globocon 2022), and the third most common cancer among both sexes [1]. India accounts for almost one-fourth of all cervical cancer deaths worldwide, with an estimated 77,348 fatalities and 123,907 new cases in 2020 (Globocon 2022). The distribution of cervical cancer varies widely; approximately 90% (342000) of deaths caused by cervical cancer occurred in low- and middle-income countries. The major etiological factor of CC is Human Papilloma Virus (HPV), including other environmental and genetic factors. High-risk HPV DNA is found in 99.7% of invasive cervical carcinomas [2]. One of the major obstacles of CC is that the majority of women are diagnosed very late, when the disease has already progressed to an advanced malignant stage. Hence, it is necessary to understand how epithelial cells acquire a mesenchymal phenotype that promotes an advanced malignant stage of cervical cancer.

The conversion of epithelial to mesenchymal cells is a multi-step process known as Epithelial-Mesenchymal Transition (EMT). Previous research has shown that extracellular signals, such as extracellular matrix elements and soluble growth factors like TGF-*β* and EGF, can trigger EMT, which is a major culprit of cancer progression [3–5]. Gene expression analysis, along with in silico data analysis, is used frequently to better understand the molecular mechanisms of cancer progression. Thus, the present study aims to investigate the molecular mechanisms of CC to identify the novel prognostic biomarkers associated with EMT.

In the present study, we have first identified the differentially expressed genes (DEGs) in CC using the GEO2R tools using microarray datasets derived from the GEO database with Accession no. GSE26511 and GSE67522, GSE9750. Then DEGs were subjected to Gene Ontology (GO), pathway enrichment analysis using Kyoto Encyclopedia of Gene and Genome (KEGG), and database for Annotation Visualization and Integrated Discovery (DAVID). In order to examine the Protein–Protein Interaction (PPI) among the DEGs, we created a protein–protein interaction (PPI) network. Next, we used Cytoscape to determine the core and hub genes among the DEGs. Furthermore, the significance of the genes and their impact on the prognosis in CC was evaluated using the Kaplan Meier Plotter Online database on the core DEGs (*P* < 0.05). Further, Gene Expression Profiling Interactive Analysis (GEPIA) was used to confirm the degree to which the expression between CC tissues and healthy cervical tissues differs. Our research may help to find out some potential biomarkers and new therapeutic targets to improve the clinical diagnosis and treatment of CC.

## Materials and methods

### Microarray data retrieval and analysis

We retrieved the microarray datasets from the GEO database (https://www.ncbi.nlm.nih.gov/gds): GSE26511, GSE67522, and GSE9750for gene expression analysis in CC and normal cervical specimens. The GPL96 platform ([HG-U133A] Affymetrix Human Genome U133A Array) was utilized by GSE9750. GSE67522 uses platform GPL10558 Illumina Human HT-12 V4.0 expression bead-chip, whereas the GSE26511 dataset was reliant on platform GPL570 ([HG-133_Plus_2] Affymetrix Human Genome U133 Plus 2.0 Array). Microarray datasets of Accession GSE26511, GSE67522, and GSE9750 include 39, 42, and 66 samples, respectively. The GSE26511 dataset represents the data of patients with early-stage cervical cancer, including 19 samples of patients with positive lymph nodes and 20 samples of patients with negative pelvic lymph nodes. Dataset 67,522 represents 11 samples with HPV negative type and 31 samples of HPV positive type, containing 20 samples of Squamous Cell Carcinoma (SCC) and 11 with normal phenotype. GSE9750 contains 9 samples from different Cell lines (HeLa, SiHa, ME-180, C4-I, CaSki, C-33 A, HT-3, SW756, MS751), 9 samples of normal cervical epithelium, 15 of normal cervix, and 33 samples of Cervical Cancer.

### Gene expression profiling analysis and for differentially expressed genes (DEGs)

DEGs between CC tissues and normal cervical tissues were identified by the GEO2R online (https://www.ncbi.nlm.nih.gov/geo/geo2r/) tool, with logFC > 0.7 and adjusted p value < 0.05 were considered significantly up-regulated, and logFC <−0.7 and adjusted p value < 0.05 were considered significantly down-regulated. The Venn diagram was carried out via Funrich 3.1.1 software to identify common genes among all three datasets, and a Volcano Plot was made using GraphPad Prism9 Software to show the significant Differentially Expressed Genes for GSE9750.

###  Identification of EMT-related genes in cervical cancer

From the list of significantly DEGs, common genes related to EMT in cervical cancer progression were identified using dbEMT 2.0 (http://dbemt.bioinfo-minzhao.org/) for further analysis. The dbEMT 2.0 database includes 1184 human EMT-associated genes curated from 2665 PubMed abstracts, comprising 1011 protein-coding genes and 173 non-coding RNAs.

### Gene ontology and pathway enrichment analysis

DAVID (https://david.ncifcrf.gov.) is a bioinformatics database created to identify the biological roles of a large number of genes or proteins. We used ‘Enrichr’, a powerful online tool for Gene Ontology that is a standardized method of classification that identifies the distinct biological roles of genes classified into Biological Process (BP), Molecular Function (MF), and Cellular Component (CC). KEGG is a collection of five manually curated databases dealing with genomes, biological pathways, diseases, drugs, and chemical substrates. DAVID and Enrichr were performed to analyze the enrichment of GO and KEGG pathways of DEGs (*P* < 0.05).

###  Protein–protein interaction (PPI) analysis

An online database called Search Tool for the Retrieval of Interacting Genes (STRING) is used to assess PPIs. STRING was utilized in order to look into any potential protein linkages among these DEGs to see the interaction network of genes, and in addition, Cytoscape was used to display the network of gene interactions and to find the Hub genes.

###  Survival analysis and expression of hub genes

A web-based application called the Kaplan-Meier plotter can be used to assess the impact of many different genes on survival using the GEO, EGA, and TCGA databases. The hazard ratio (HR) with 95% confidence intervals was calculated and displayed on the plot along with the log-rank P value. The Gene Expression Profiling Interactive Analysis (GEPIA) website was utilized to verify the expression of these DEGs. Box plot and Stage plot were made to display the variation, degree of dispersion, and skewness of the genes with different stages in case of normal and cervical cancer samples.

## Results

### Identification of DEGs for EMT in cervical cancer

**Fig. 1:**
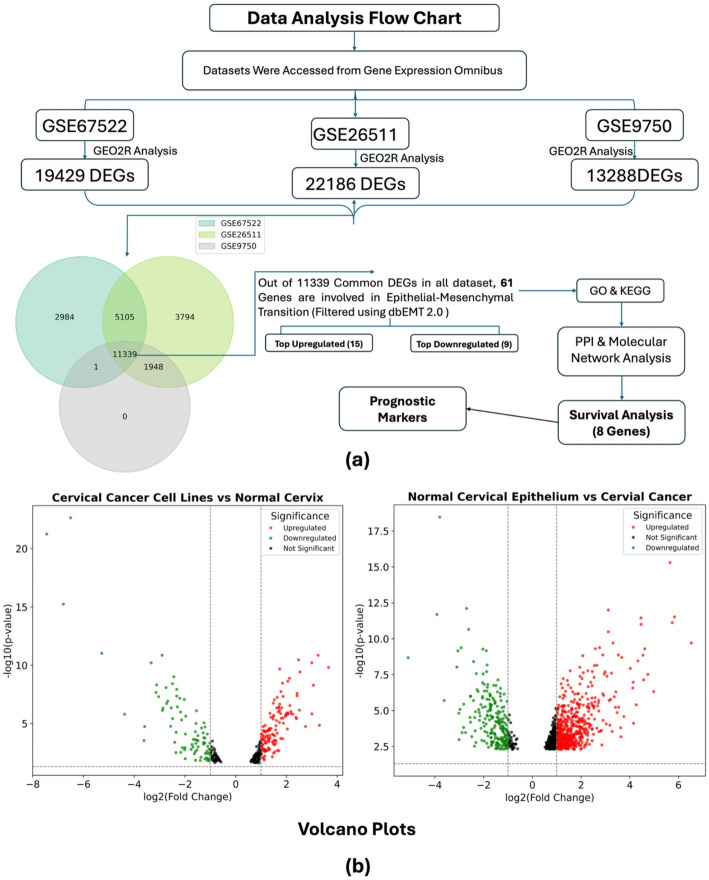
**a** Data Analysis Workflow and Venn diagram representing the overlaps between three datasets (GSE26511, GSE67522, and GSE9750) to get common DEGs in three different colours representing different datasets made via Funrich software. **b** Volcano Plot showing Differentially Expressed Genes in GSE9750 dataset, Cervical cancer Cell lines VS Normal Cervix and Normal Cervical EPithelium VS Cervical Cancer

 To identify genes that are related to EMT in CC progression, firstly, we explored DEGs that are possibly involved in the EMT from normal to cervical cancer progression. We downloaded microarray data from NCBI-GEO having Accession nos. GSE26511, GSE67522, and GSE9750 with 39, 42, and 66 samples, respectively. By using the GEO2R online tool, we have analyzed the microarray data (p-value = 0.05, Benjamini & Hochberg (False discovery rate) and logFC > 0.7), and extracted 54,675, 42,670, and 12,260 DEGs from GSE26511, GSE67522, and GSE9750, respectively. Subsequently, a Venn diagram was made using “Funrich” software to get the common DEGs existing in all three datasets. As can be seen from Fig. 1a, a total of 11,339 DEGs were identified as common.

Among the 11,339 common differentially expressed genes (DEGs) identified across all three profile datasets, 24 genes (15 upregulated and 9 downregulated) were consistently found to be associated with epithelial–mesenchymal transition (EMT) regulation in cervical cancer tissues compared to normal tissues, as shown in Table 1 and Table S1 within Supplementary file. Furthermore, 61 genes from the pool of 11,339 common DEGs were linked to the EMT pathway involved in cervical cancer progression (Table 2; Table EMT within Supplementary file).

In addition, the Volcano Plot for GSE9750 accession was obtained for Cervical Cancer cell lines Vs Normal Cervix and Normal Cervical epithelium Vs Cervical Cancer shown below where x axis representing log2 fold change and y axis representing –log10 (p value) depicting the significant (*p* < 0.05) up-regulated genes in red, down-regulated genes in green whereas the non-significant genes are in black shown in Fig. 1b.

**Table 1:** Represents 15 up-regulated genes (logFC > 0.7) and 8 down regulated genes (logFC<−0.7)

DEGs	Gene terms
Up-regulated genes	VIM, FN1, DCN, CAV1, CD24, CDH11, CXCL12, ZEB1, ZEB2, TGFB1, TWIST, SFRP4, IL6, WNT5A, CDH2
Down regulated genes	CDKN2A, MMP3, PKP3, CLDN7, SIX1, MMP9, KRT8, MMP14, TP63

**Table 2:** Represents list of 61 EMT associated genes identified from 11339 common DEGs in cervical cancer progression

BRD2, BRD4, CDH1, CDH2, TWIST, ZEB1, ZEB2, MMP9, MMP2,MMP3, VIM, FN1, SMAD2, SMAD4, SNAI1, SNAI2, WNT5A, LOXL2, TGFB1, THBS1, PDGFRB, IL6, CTGF, CD24, FAP, COL6A3, ID2, TP63, POSTN, SFRP4, LUM, CXCL12, DCN, HTRA1, SIM2, PDCD1, AQP5, CDKN2A, ONECUT2, SIX1, MYBL2, PKP3, KRT18, KRT14, BDNF, CAV1, ESRP1, SPARC, CD44, MMP14, MMP16, CDH11, FGF2, FGFR1, TNC, COL3A1, CLDN7, CLDN4, KRT8, JUP, OCLN	

### Gene ontology (G.O) and KEGG pathway analysis of DEGs for EMT

**Fig. 2:**
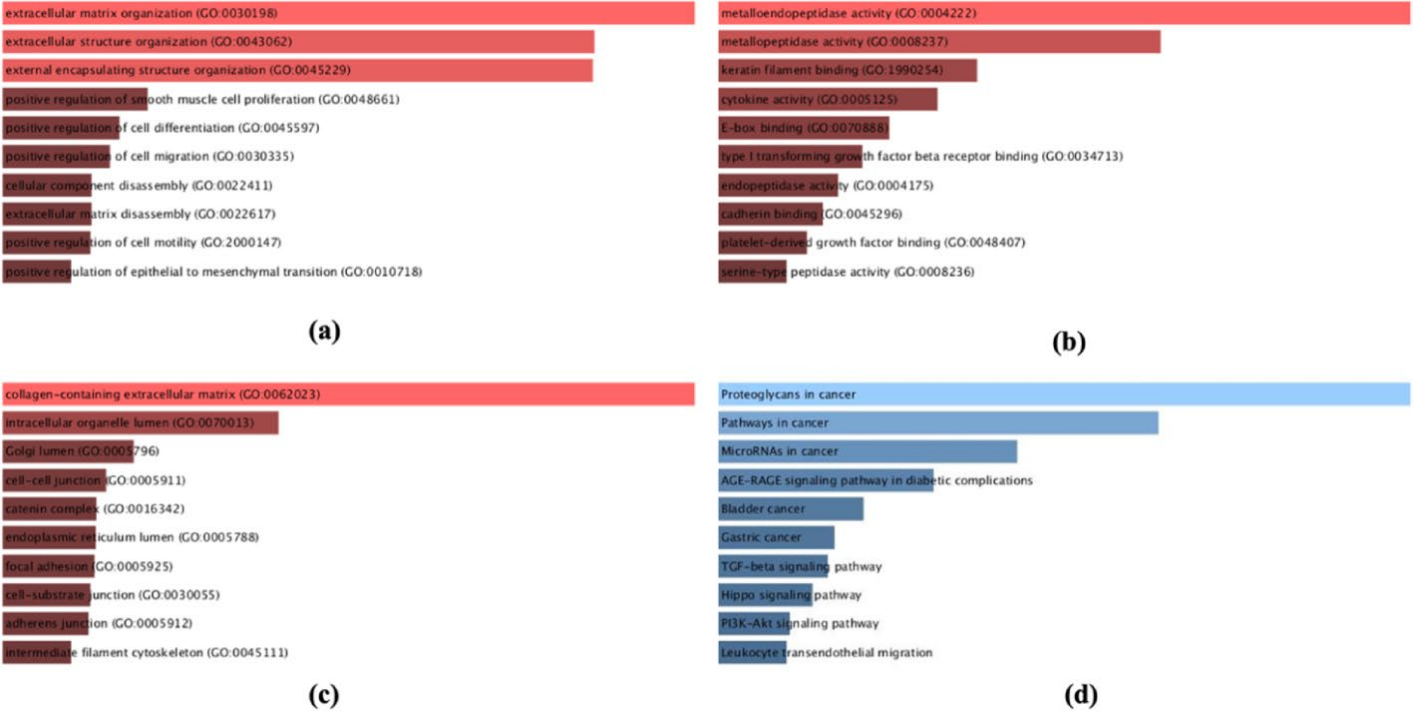
Results of the GO term and KEGG pathway enhancement studies carried out with Enrichr. The top 10 that are enriched in KEGG pathway, cellular component, biological process, and molecular function. The x-axis addresses the number of genes and the y-axis addresses the (**a**) BP (**b**) MF (**c**) CC and (**d**) KEGG Pathway names

Using Enrichr we identified the gene ontology of the 61 DEGs involved in EMT in cervical cancer progression and the bar chart is obtained. The G.O analysis indicates that (1) for Biological Process (BP), the genes are enriched in regulation of cell proliferation, differentiation, mobility and migration as well as in EMT. (2) For molecular function (MF), the genes were enriched in metallopeptidase activity, TGF-β receptor binding and transcription regulator activity. (3) for Cellular component (CC), the genes are enriched in cellular anatomical entity and protein-containing complex as mentioned in Figure (Fig. 2).

**Fig. 3:**
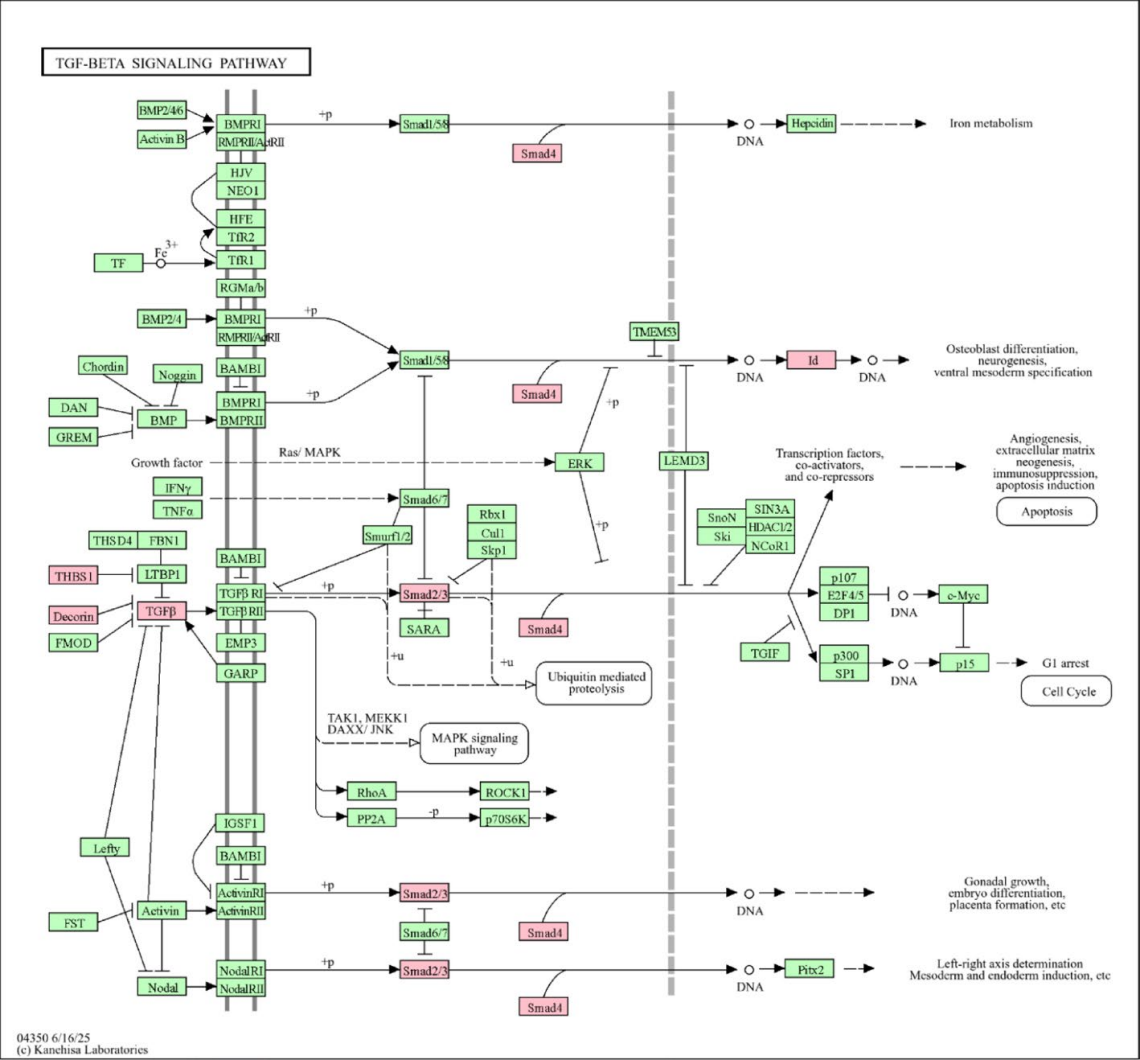
KEGG pathway for TGF-β Signaling (6 DEGs are star-marked in the signaling pathway)

To explore the pathway associated with the common DEGs of EMT in Cervical Cancer, the DEGs were further analyzed using the DAVID web tool, and the result shows that the common 61 DEGs associated with EMT are found to be linked with 44 KEGG pathways. To know the DEGs involved in the TGF-β pathway, we explored the KEGG pathway for TGF-β signaling shown in Fig. 3 and found 6 genes out of 61 common DEGs are found to be linked with the pathway, they are SMAD2, SMAD4, ID, Decorin, THBS1, and TGFβ.

### Protein–protein interaction (PPI) and molecular network analysis

**Fig. 4:**
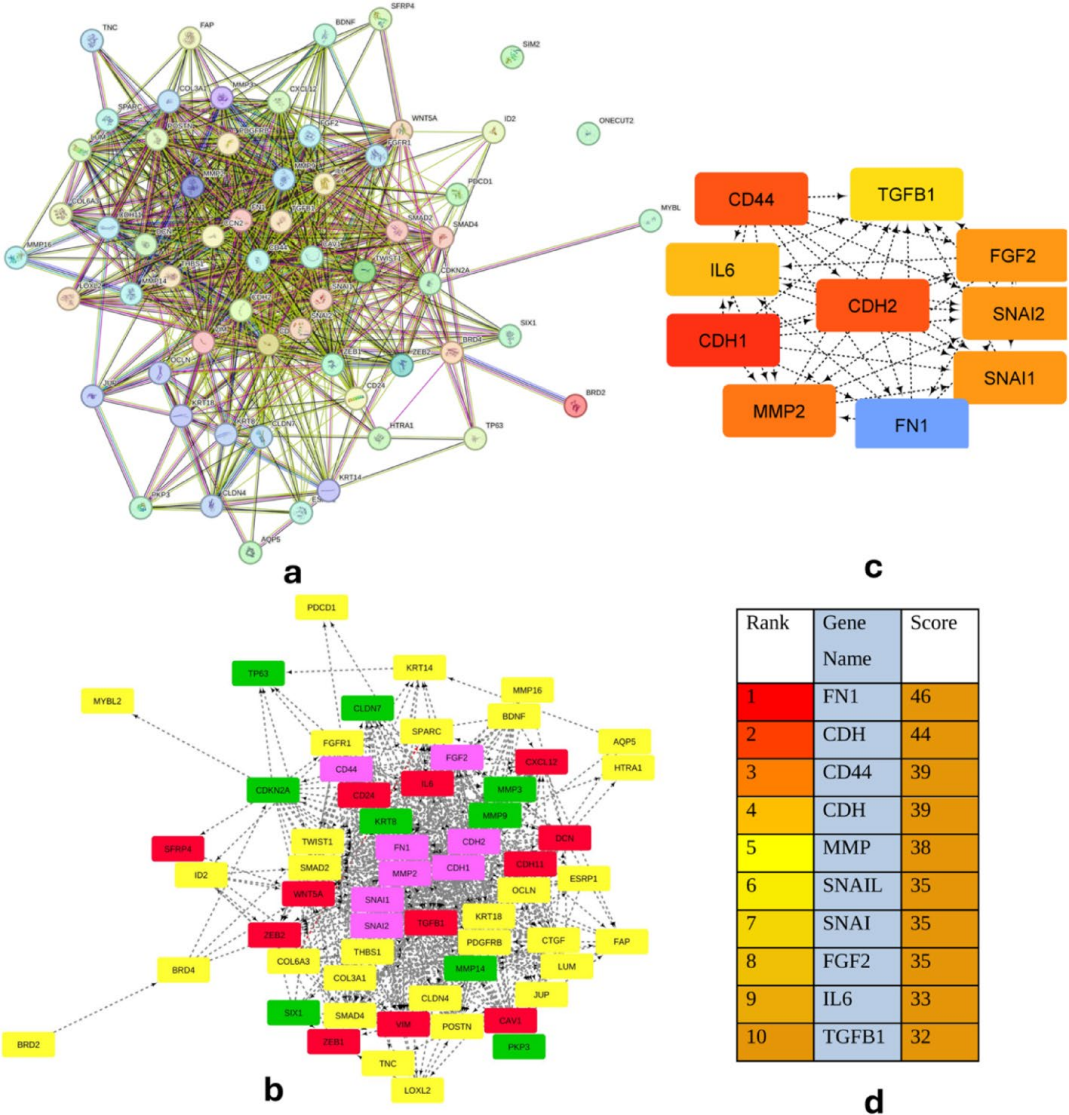
**a** By using the STRING database, the PPI network of common DEGs was created, including 61 common DEGs in EMT in CC, where the nodes indicate the proteins and the lines represent the interactions among proteins. **b** Module Analysis through Cytoscape Software showing up-regulated (red), down-regulated (green), and hub genes (pink) (**c**) subnetwork of top 10 hub genes from PPI network using Cytoscape software (**d**) top 10 interactions ranked by degree method

PPI network of the 61 DEGs involved in EMT in cervical cancer obtained from STRING shows a large network of genes, to identify the up-regulated, down-regulated, and more specifically the hub genes that play a major role in EMT in Cervical Cancer. The PPI network was downloaded and converted to a TSV file format. The tsv file was then added to the Cytoscape software’s Cyto-Hubba plug-in, which is included in version 3.8.2, and the y files’ organic layout network image was designed on Cytoscape for a clear visualization of the gene network. The Cytoscape network image shows the up-regulated gene in red, the down-regulated gene in green, and all other genes in yellow. The hub genes are marked in pink, which are 8 in number, with the highest interaction indicating the genes playing a critical role in EMT in cervical cancer progression. The highest degree score from the protein–protein interaction result was used to identify the hub genes. The hub gene clustering nodules are found using Cytoscape’s MCODE (molecular complex detection) plug-in. The arrow indicates the direction of the target gene from its source gene. FN1, MMP2, CDH1, CDH2, CD44, FGF2, SNAI1, and SNAI2 are found to be the hub/critical genes associated with EMT in cervical cancer, as depicted in Fig. 4.

### Survival analysis of the key genes

**Fig. 5:**
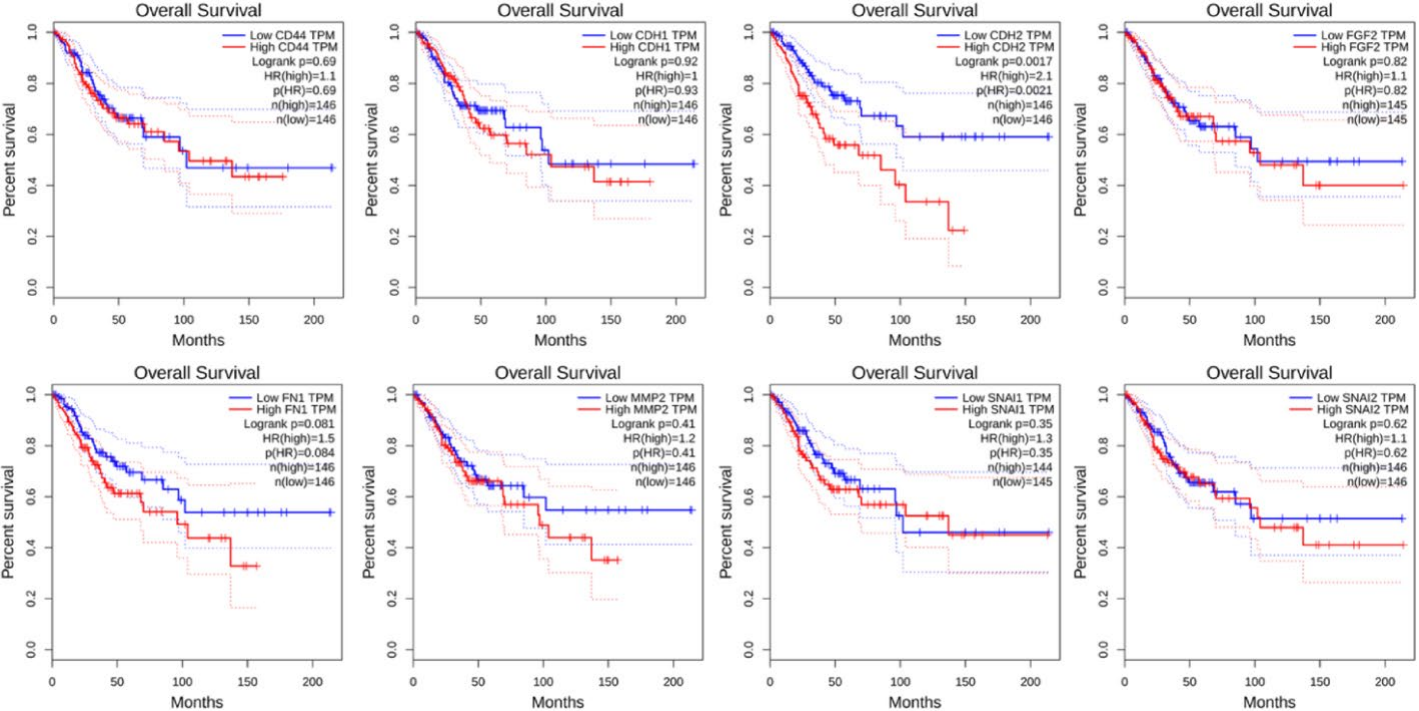
The overall Survival curve predicting the prognostic value of the genes

**Fig. 6:**
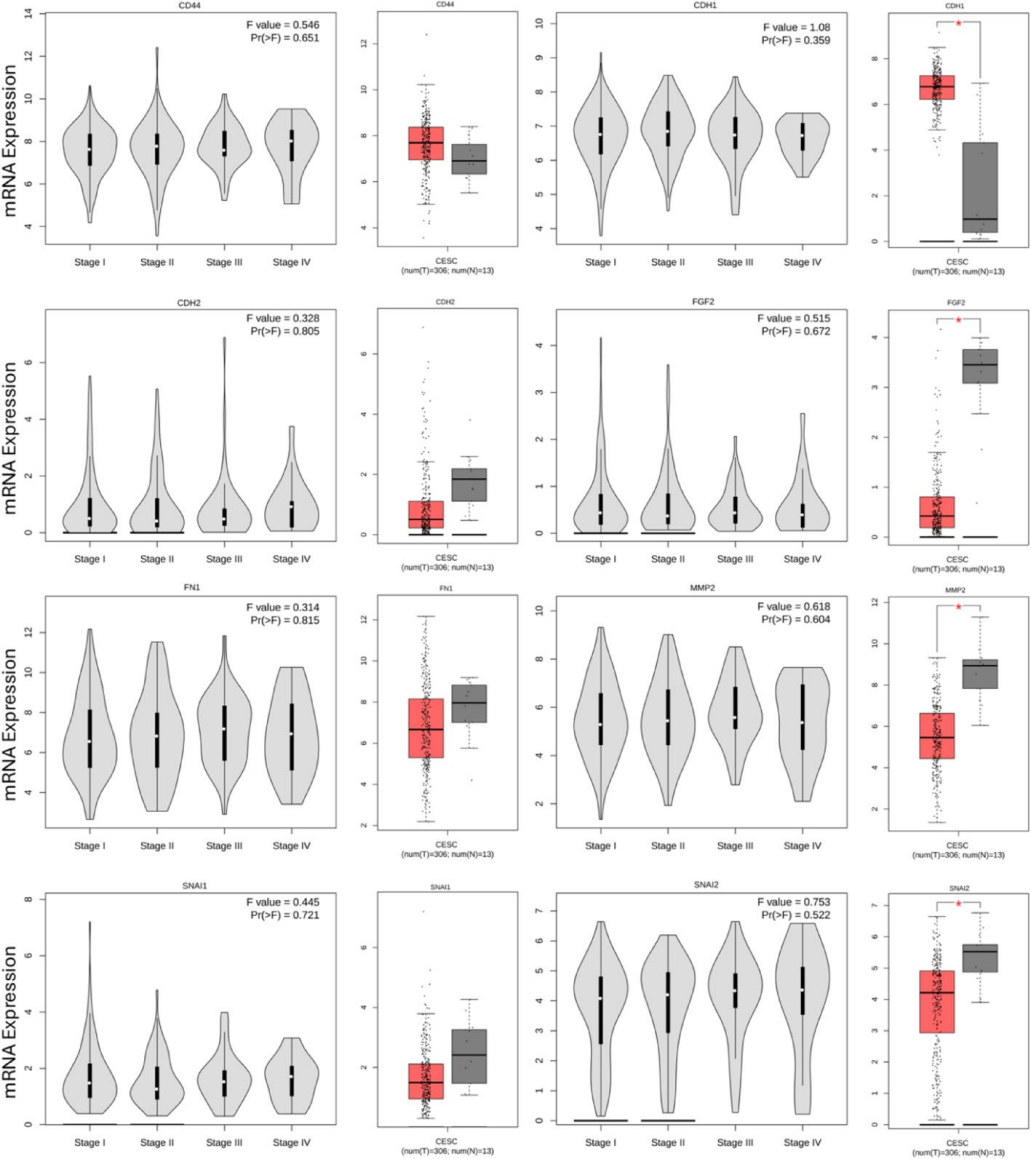
The box plot and stage plot showing the gene expression in normal and cervical cancer tissues (The red star denotes the significant expression of a particular gene in CESC)

The prognostic significance of hub genes was examined by GEPIA, which is a web server for interactive analysis and profiling of gene expression in cervical cancer patients’ data. The patients were divided into high and low expression groups; the prognostic value of the genes predicts the overall survival in cervical cancer patients. The KM plotter survival graph and box plot expression analysis of biomarker genes demonstrated the prognostic significance of eight hub genes (FN1, MMP2, CDH1, CDH2, CD44, FGF2, SNAI1, and SNAI2) that play an important role in EMT progression in CC, as shown in Figs. 5 and 6.

## Discussion

Cervical cancer is the most common gynecological malignancy in terms of both incidence and mortality worldwide. It has an impact on the uterine cervix’s cell lining, which goes through a sequence of Cervical Intraepithelial Neoplasia (CIN) alterations to develop into invasive cervical cancer. Often, precancerous cell turns into cancerous cells and take a long time to develop into invasive cancer. Thus, early diagnosis of cervical cancer using biomarkers can help prevent cervical cancer progression. Only chemotherapy, radiation therapy, and surgery are widely used to treat cervical cancer in the current scenario, which is not enough and not effective to save the lives of the patients [6]. Hence, studies about the molecular mechanisms underlying the pathogenesis of cervical cancer progression and its associated genes are essential to discover critical biomarkers. Despite a good early prognosis of the CC, many patients still die due to metastasis, drug resistance, and recurrence of the diseases, where EMT play a very crucial role. Since EMT is implicated in cancer metastasis, identifying a molecular marker using in silico datasets of CC may provide evidence to find a molecular marker in cervical cancer metastasis. Other studies have also mined the GSE9750 datasets [7, 8] in various signaling pathways linked with cervical carcinoma and potential drugs used. Some recent studies on GSE64217 datasets examined the expression levels of IGF2PB3 and PTPRZ1 genes, suggesting a marker for cervical cancer prognosis in CC samples and normal samples [9]. Some other studies have shown DSG2, PLOD2, ANLN, AURKA, and AR genes might be key genes associated with cervical cancer progression [10, 11]. To date, no study has yet examined the genes associated with EMT in cervical cancer progression, metastasis, and their link to molecular pathways.

In this study, we have analyzed three microarray datasets (GSE9750, GSE7803, and GSE26511) having samples from normal cervix, CIN, SCC, and Cervical Cell lines to find out differentially expressed genes (DEGs). We have found 11,339 common differentially expressed genes, among all 15 and 8 genes significantly upregulated and downregulated, respectively. Interestingly, our work highlights 61 common genes that are associated with EMT in cervical cancer. Further, Gene ontology (GO) analysis shows the 6 genes are enriched in molecular function, biological process, and cellular component mainly linked to SMAD4-TGF-β signaling pathway, one of the important pathways involved in EMT. Our study has also identified 8 hub genes having the highest degree of interactions using protein–protein interaction and its visualization by Cytoscape 3.8.2. As per the survival analysis in cervical squamous cell carcinoma (CESC), CDH2 (N-cadherin) shows a significant overall survival association with a p-value of 0.0017 and a hazard ratio (HR) of 2.1, indicating its strong prognostic relevance in disease progression. Similarly, FN1 (fibronectin) is significantly associated with disease-free survival (*p* = 0.043, HR = 1.8), confirming its role in promoting tumor recurrence through epithelial-mesenchymal transition processes. CD44, a well-recognized cancer stem cell marker, contributes to epithelial–mesenchymal transition (EMT) by enhancing cellular plasticity, migration, and invasion through its interactions with hyaluronan and the regulation of key EMT transcription factors [12]. CDH1 (E-cadherin) serves as a critical epithelial marker whose loss weakens cell–cell adhesion, thereby facilitating EMT and promoting cancer cell dissemination [13]. FGF2 (fibroblast growth factor 2) drives EMT by activating signaling cascades such as TGF-β, which in turn increase the expression of mesenchymal markers and matrix-remodeling enzymes [14]. MMP3 (matrix metalloproteinase 3) supports EMT by degrading extracellular matrix components, enabling greater tumor cell invasion and metastasis. Among the transcriptional regulators, SNAI1 (Snail) acts as a master repressor of epithelial markers—particularly CDH1 [15] thus orchestrating the EMT program essential for metastasis, while SNAI2 (Slug) similarly represses epithelial genes and governs cellular plasticity during EMT [16], contributing to cancer progression and therapeutic resistance. This indicates these genes are critically associated with EMT progression that can be further use as diagnostic and prognostic markers.

However, the 8 hub key genes identified in the current study associated with EMT in cervical cancer progression must be validated at a laboratory level, either using ex vivo tissue or in vitro cell line models. Furthermore, the data generated in the current study only provide a starting point for investigation aimed at delineating the molecular mechanism of EMT in cervical cancer progression.

## Limitations and future prospects

In the present study, we identified eight key hub genes CDH1, CDH2, MMP2, CD44, FN1, FGF2, SNAI1, and SNAI2 that are associated with epithelial-mesenchymal transition (EMT). Among these, CDH2 and FN1 showed a significant correlation with EMT progression and may serve as potential diagnostic and prognostic biomarkers for cervical cancer. As these findings are based on preliminary in silico analyses, further validation is necessary. Therefore, future studies will focus on confirming these biomarkers through molecular analyses using mouse models, in vitro cell lines, and clinical samples from cervical cancer patients. Such validation could contribute to the development of personalized therapeutics and enhance targeted treatment strategies, ultimately improving the survival rate of cervical cancer patients.

## Conclusion

Cervical cancer remains one of the most common gynecological malignancies worldwide, with a significant impact on female health due to its high incidence and mortality rates. It progresses through a well-characterized sequence of cellular changes, from cervical intraepithelial neoplasia (CIN) to invasive cancer, often over prolonged periods, which provides a critical window for early diagnosis and intervention [2]. Current treatment approaches, predominantly chemotherapy, radiation, and surgery, have limitations and are insufficient to substantially improve survival, particularly due to issues such as metastasis, drug resistance, and recurrence. The epithelial-mesenchymal transition (EMT) plays a pivotal role in promoting these aggressive tumor behaviors. Therefore, understanding the molecular mechanisms driving EMT and identifying associated biomarkers is essential for better prognosis and therapy in cervical cancer.

This study analyzed three microarray datasets to identify differentially expressed genes linked to cervical cancer progression, highlighting 61 genes associated with EMT. Gene ontology analysis identified key involvement of these genes in pathways, including TGF-β signaling, which is critical in EMT regulation. Protein–protein interaction network analysis revealed 8 hub genes with significant interactions, suggesting their central role in cervical cancer progression. Among eight EMT hub genes; CDH2 (N-cadherin) showed a significant association with overall survival (*p* = 0.0017, HR = 2.1), and FN1 (fibronectin) was notably linked with disease-free survival (*p* = 0.043, HR = 1.8) (Supplementary Fig. 1), indicating their prognostic importance in cervical cancer. Additionally, other EMT-related genes, CD44, CDH1, FGF2, MMP3, SNAI1, and SNAI2, have established roles in enhancing cellular plasticity, invasion, and metastatic potential by orchestrating EMT processes [12–16]. Collectively, these findings emphasize the critical contribution of EMT-associated genes to cervical cancer progression and underscore their potential as diagnostic and prognostic biomarkers, as well as therapeutic targets to improve patient outcomes [17].

This integrated analysis offers valuable insights into cervical cancer biology and opens avenues for developing EMT-targeted interventions to address metastasis and therapy resistance, which remain major challenges in cervical cancer management. In conclusion, the use of in silico gene data mining study only provides an excellent framework for the initial identification of key 8 hub genes associated with EMT and it needs to be validated using ex vivo tissue, mice model, and in vitro cell lines of cervical cancer.

## Supplementary Information

Supplementary Material 1.

Supplementary Material 2.

## Data Availability

All datasets analyzed in this study were obtained from publicly available repositories. Specifically, the gene expression datasets are available in the Gene Expression Omnibus (GEO) database, which is hosted by the National Center for Biotechnology Information (NCBI), U.S. National Library of Medicine (https://www.ncbi.nlm.nih.gov/geo/). The corresponding GEO accession numbers and direct web links for the datasets used for downstream analysis are GSE26511 (https://www.ncbi.nlm.nih.gov/geo/query/acc.cgi? acc=GSE26511), GSE67522 (https://www.ncbi.nlm.nih.gov/geo/query/acc.cgi?acc=GSE67522),andand GSE9750 (https://www.ncbi.nlm.nih.gov/geo/query/acc.cgi?acc=GSE9750). The detailed datasets analyzed during the current study are available with the corresponding author. In the future, it will be made available on reasonable request.
