# Supplementary figures and images for: Genes associated with epithelial mesenchymal transition (EMT) in cervical cancer progression
Source: Discov Oncol. 2025 Nov 20;16:2245. doi: 10.1007/s12672-025-04011-y (PMC12748436; doi:10.1007/s12672-025-04011-y)

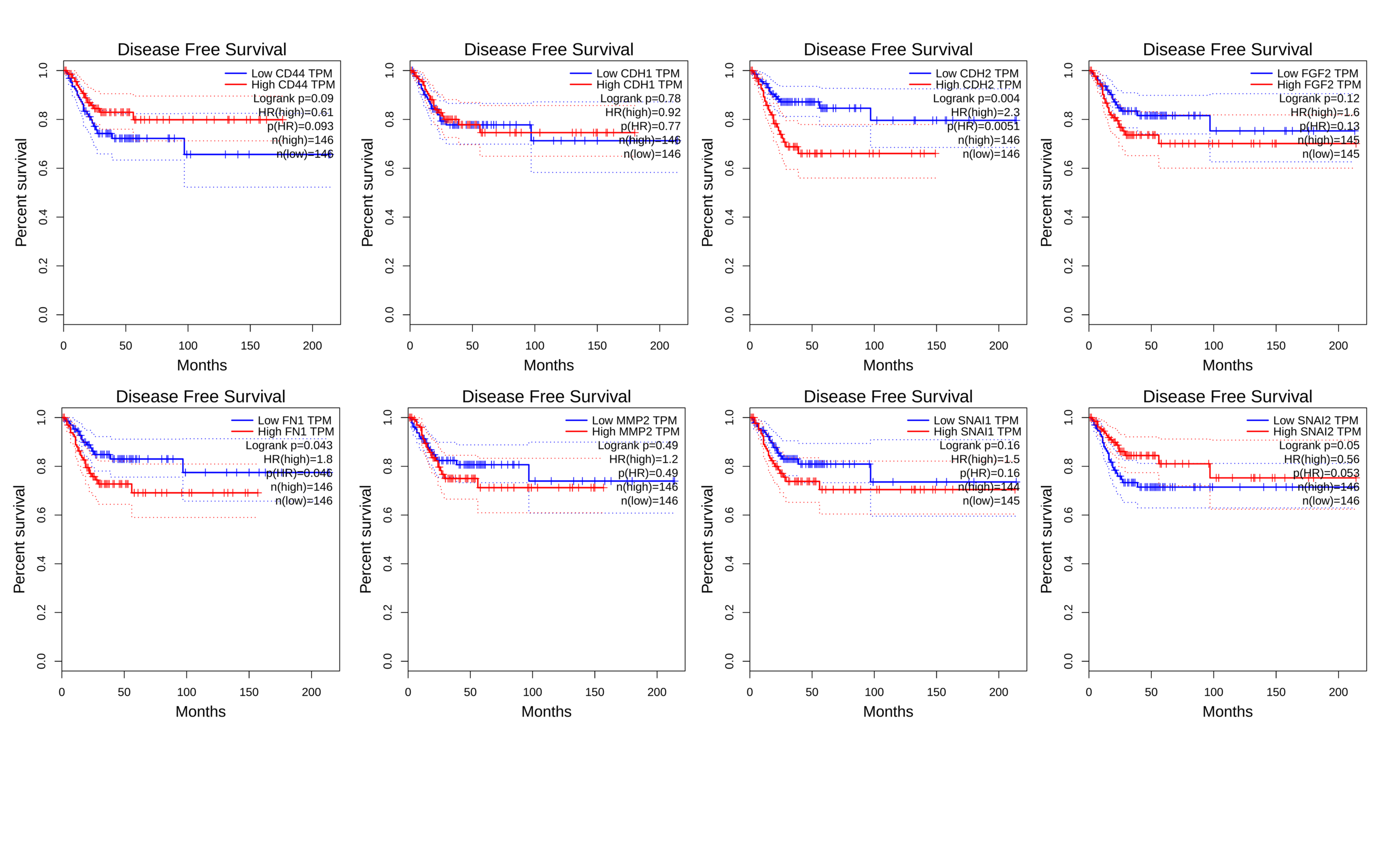

Supplement: Supplementary file 1 — Supplementary Material 1. [file 12672_2025_4011_MOESM1_ESM.png]
